# Polyethyleneimine-Based Lipopolyplexes as Carriers in Anticancer Gene Therapies

**DOI:** 10.3390/ma15010179

**Published:** 2021-12-27

**Authors:** Julia Jerzykiewicz, Aleksander Czogalla

**Affiliations:** Department of Cytobiochemistry, Faculty of Biotechnology, University of Wroclaw, Fryderyka Joliot-Curie 14a, 50-383 Wroclaw, Poland; jerzykiewicz.julia@gmail.com

**Keywords:** lipopolyplexes, polyethyleneimine, nucleic acids, lipids, liposomes, non-viral vectors, gene therapy, cancer

## Abstract

Recent years have witnessed rapidly growing interest in application of gene therapies for cancer treatment. However, this strategy requires nucleic acid carriers that are both effective and safe. In this context, non-viral vectors have advantages over their viral counterparts. In particular, lipopolyplexes—nanocomplexes consisting of nucleic acids condensed with polyvalent molecules and enclosed in lipid vesicles—currently offer great promise. In this article, we briefly review the major aspects of developing such non-viral vectors based on polyethyleneimine and outline their properties in light of anticancer therapeutic strategies. Finally, examples of current in vivo studies involving such lipopolyplexes and possibilities for their future development are presented.

## 1. Introduction

In recent years, intensive research on genetic aberrations that underlie various cancers has revealed numerous potential molecular targets for gene therapies. The introduction of exogenous nucleic acids in the form of plasmid vectors, antisense oligonucleotides or mRNA, to name a few, enables complete elimination of pathological cells (for example, using ‘suicide genes’) and/or limitation of their proliferation (by silencing expression of oncogenes or restoring function of key suppressors) [1]. Most recently, a broad range of non-coding RNA molecules have been shown to fine-tune not only gene expression but also genome remodeling, thus becoming new tools to modulate disease-related physiological processes [2,3].

Nevertheless, the efficient delivery of therapeutic DNA or RNA to the target cells still remains a great challenge. The instability of these molecules in serum and their lack of target specificity necessitates at least modification of the sugar-phosphate backbone to make them less susceptible to naturally occurring nucleases (for example, replacement of phosphate bonds with phosphorothioate ones in various oligonucleotide drugs, such as mipomersen and nusinersen, both approved by the U.S. Food and Drug Administration (FDA)) or application of an appropriate carrier [4,5]. Presently, viral vectors constitute a widespread platform for delivery of nucleic acids. Viruses can be reprogrammed to deliver genetic drugs into target cells while minimizing their pathogenicity. However, their high immunogenicity and mutational potential raise some important concerns [6,7,8]. For example, lentiviral carriers may integrate carried genetic material into host genome in a random location, which could lead to accidental creation of an oncogene [9]. Moreover, viral vectors are challenging to manufacture in a cost-effective way at a commercial scale, which stems mainly from their intrinsic characteristics as biological drugs [4,10]. Thus, non-viral alternatives are sought with the aim of ensuring a comparatively high level of transfection and simultaneous increased biocompatibility [9,11,12]. The latter type of vectors also has their own, additional advantages, including higher capacity towards large nucleic acid molecules, excellent reproducibility, and relatively low costs of manufacturing and scaling up the production [13]. Additionally, they are relatively easy to optimize in terms of both the delivered genetic material and the carrier itself, which is supposed to protect and precisely target its cargo [14]. Among various types of non-viral gene delivery systems, those based on either cationic lipids or polymers are most prominent.

Lipoplexes, i.e., complexes of cationic lipid-containing liposomes with nucleic acids, are routinely used as transfecting agents (e.g., Lipofectamine) [15]. In recent years, the number of preclinical studies and clinical trials involving this type of carrier has increased. This yielded, for example, patisiran (Onpattro), a drug against polyneuropathy based on a modified siRNA particle enveloped in a lipid nanocarrier, the first drug of its kind approved, in 2018, by the FDA. This therapeutic nucleic acid is delivered with the help of phospholipids, ionizable cationic and PEGylated lipids with addition of cholesterol. After intravenous administration, it localizes primarily in the liver, where high levels of its molecular target occur [16]. Patisiran presents excellent effectiveness even after administration of a single dose. However, when considering lipoplexes in a more general context some limitations, such as high cytotoxicity at high concentrations, relatively low transfection efficiency, and difficulties with size optimization, present barriers to the broad use of similar carriers in other therapies [17].

Polyplexes, on the other hand, are complexes of nucleic acids with polycationic polymers. Among various positively charged molecules available, the ones broadly researched are: poly-L-lysine (PLL), dendrimer poly(amidoamine) (PAMAM), and polyethylenimine (PEI), with the latter being the most promising. Despite a plethora of studies focused on modification of such polycations to ensure their biodegradability, higher specificity, or stability, polyplexes still face similar difficulties as lipoplexes, with low transfection levels being the primary obstacle to therapeutic use [18]. It is worth underlining that, even in in vitro conditions, prolonged exposure of cells to PEI/DNA complexes may significantly impair cellular proliferation and viability [19].

However, in recent years, a new generation of carriers, called lipopolyplexes, has come into play. They combine polyplexes and lipid vesicles, drawing benefits from both systems. Liposomes themselves, due to their biocompatibility and versatility, have long since been used in clinical practice, over and above the classical anti-cancer therapies [20]. A wide range of available lipids capable of spontaneous formation of a stable bilayer and the ease of its modification permit the preparation of a carrier tailored for individual therapeutic needs, which, in the context of lipopolyplexes, is described in detail below. Their chemical composition also has a strong impact on the stability of the formulation, both during storage and when introduced to the bloodstream. Among such promising, non-viral carriers are polyethyleneimine-based lipopolyplexes, which are the subject of this article.

## 2. Polyethyleneimine-Based Lipopolyplexes as Nucleic Acid Carriers

**Figure 1:**
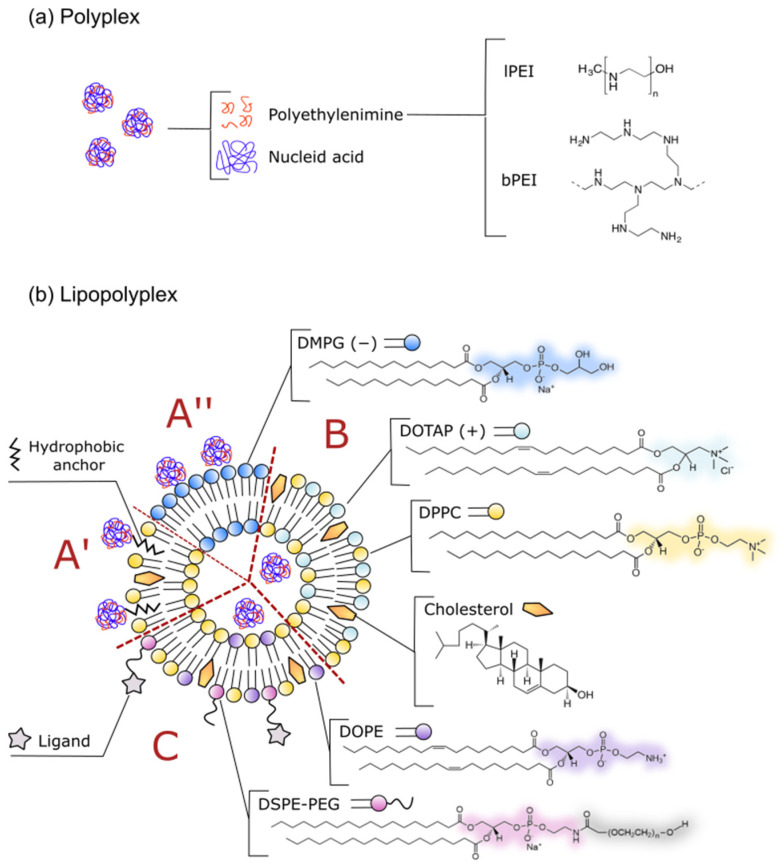
Structure and components of various polyethyleneimine-based lipopolyplex carriers for gene delivery. A’—Reversed lipopolyplexes with polyethyleneimine bound covalently to hydrophobic anchor (e.g., triamcinolone acetonide) embedded in a neutral lipid bilayer; A”—reversed lipopolyplexes composed of anionic lipids that enable docking of positively charged polyplexes based on polyethyleneimine; B—“classical” lipopolyplexes with the addition of cationic lipids; C—lipopolyplex with targeting ligands (e.g., antibodies, transferrin, aptamers) conjugated to the surface via polyethylene glycol (PEG) modified lipids. bPEI—branched polyethyleneimine; DMPG—1.2-dimyristoyl-sn-glycero-3-phospho-(1′-rac-glycerol) (sodium salt), negatively charged; DOPE—1.2-dioleoyl-sn-glycero-3-phosphoethanolamine; DOTAP—1.2-dioleoyl-3-trimethylammonium-propane (chloride salt), positively charged; DPPC—1.2-dipalmitoyl-sn-glycero-3-phosphocholine; DSPE-PEG—1.2-distearoyl-sn-glycero-3-phosphorylethanolamine conjugated with polyethylene glycol chains; lPEI—linear polyethyleneimine.

The structure of lipopolyplexes based on polyethyleneimine varies depending on formulation; exemplary components and the schematic layout of such carriers are shown in Figure 1.

The required component of such lipopolyplexes, except therapeutic nucleic acid, is polyethyleneimine (PEI)—a relatively easy to manufacture (and inexpensive) polycation, used to introduce nucleic acids into eukaryotic cells in vitro since 1995 [21]. PEI is usually synthesized by acid catalyzed ring-opening polymerization of aziridine monomers resulting in random branched polymers (bPEI), while linear PEIs (lPEI) are the products of polymerization of 2-substituted 2-oxazoline monomers [22]. While lPEI have secondary amines in the backbone and primary amines at the polymer ends, bPEI contain primary, secondary and tertiary amine groups in approximate ratio 1:2:1, which strongly influences buffering capacity of the polymer [23]. Both forms of PEI are available over a broad range of molecular weights (0.6–1000 kDa). Its major advantages originate from chain flexibility and high positive charge density (protonable amino groups in every third position), which not only allow the condensation of even large nucleic acid molecules into the complexes on the nano scale but also provide a high buffer capacity [24,25,26]. All of the above contribute to the relatively high efficiency of transfection with polyplexes, regardless of the cell cycle phase of the target [27]. Furthermore, PEI stabilizes DNA and RNA by generating electrostatic interactions with the sugar-phosphate backbone, thus protecting them against intracellular and extracellular nucleases [28]. It is also speculated that polyethyleneimine could act at low pH as a “proton sponge” [29], as its amine groups can absorb protons pumped into the endosome and lead to an increased influx of chloride ions and water. This facilitates endosome escape of polyplexes, as they enter cells via endocytosis (both caveolar and clathrin-dependent) [23,30]. However, some studies indicate that the mentioned phenomenon, which leads to differences in osmotic pressure on both sides of the endosomal membrane, may be insufficient for the effective release of polyplexes into the cytosol [31,32,33,34]. Hence, it is suspected that the direct destabilization of the negatively charged endosomal membranes by positive charge of PEI plays a greater role in this process [31]. In addition, it is suggested that polyethyleneimine may influence the further fate of the whole complex, directing it to the nucleus, as it was observed that both polyplexes and free polymer chains tend to relocate into the nucleoplasm [35]. A broad range of modifications of PEI with diverse functionalized segments were also introduced for improved transfection efficacy, pharmacokinetics, and bioavailability, which were reviewed elsewhere [26,36].

Nevertheless, condensation of nucleic acids in a complex with polyethyleneimine alone would not be sufficient to create an effective platform for systemic delivery in vivo, as mentioned in the Introduction. The main obstacle seems to be the non-specific interactions between cationic polyplexes or unbound PEI molecules with plasma proteins or components of biological membranes [37,38,39,40]. In some cases, this might trigger an immune response through the complement system, and eventually induce direct cytotoxicity, bringing the target cell down the mitochondrial-mediated apoptosis pathway [41,42,43,44,45].

However, encapsulating polyplexes with a lipid bilayer, and, therefore, creating a lipopolyplex, may be a solution providing prolonged stability after intravenous administration and diminishing the negative impact on normal cells [46]. Combination of both lipid vesicles and polyplexes shows many advantageous features as nucleic acid carrier systems and enables further improvement of their effectiveness and biocompatibility due to the overlap of superior properties of liposomal systems (for instance, lowered cytotoxicity, relatively high cellular uptake and stability) and polyplexes (e.g., efficient condensation of nucleic acids and facilitated endosomal escape) [47].

Presence of a lipid bilayer surrounding polyplexes not only limits interaction of such complexes with anionic components of serum but also prevents their spontaneous aggregation, maintaining the biological activity of the carrier stored as a suspension for longer periods of time [47,48,49]. This is usually reflected by changes of zeta potential of polyplexes after their encapsulation in liposomes [48]. However, there is still space to improve the long-term stability, especially in light of potential dependence on the lipid composition and modulation of the fusogenic capacity of PEI [38,50]. In this context, essential parameters that characterize lipopolyplexes, in analogy to other liposomal formulations, are particle size and size distribution [51]. These parameters not only define the stability of the formulation but also heavily influence in vivo performance, including cellular uptake and biodistribution [20].

The use of lipopolyplexes usually allows for much more effective transfection, both in vitro and in vivo, compared to polyplexes alone [47,52]. More specifically, lipopolyplexes present satisfactory pharmacokinetics when administered intravenously [53,54]. In the case of carriers without any specific targeting ligands on their surface, e.g., those coated with polyethylene glycol (see below), which makes them stealth carriers and thus more stable in the bloodstream [55,56], the main parameter limiting penetration of tissues is size. In anti-cancer therapies, this allows the use of a passive targeting strategy based on the occurrence of an enhanced permeability and retention effect (EPR), which refers to abnormal structure of endothelium of blood vessels and lack of lymphatics surrounding the tumor [54,57,58]. Moreover, the modular architecture of lipopolyplexes enables further modifications of their surface, including addition of targeting molecules, such as antibodies and their fragments, peptides, and proteins, aptamers, or sugar moieties, not interfering with either the structure of the polyplexes located inside of the vesicles or the mechanical properties of the lipid shell [59,60]. Receptors overexpressed at the surface of cells exhibiting compromised mechanisms of cell proliferation and/or survival regulation have become one of the most common targets, for instance, folate receptor α (FRα) or transferrin receptor 1 (TfR1) [60,61,62]. Exposure of their agonists on the surface of genetic drug carriers often leads to improvement of their internalization kinetics [60]. Such active targeting also reduces cytotoxicity to normal cells, while maintaining efficient transfection of pathological ones [54,63]. For example, cruciality of targeting in some cases was demonstrated on patient-derived glioblastoma stem-like cells, where non-functionalized lipopolyplexes were internalized to a negligible degree. In contrast, vesicles exposing on the surface fibronectin-mimetic peptide that specifically targets the α_5_β_1_ integrin (protein overexpressed in glioblastomas) presented satisfactory transfection efficiency in vitro [64]. Nevertheless, it should be taken into consideration that some ligands are specific only for cancer cells of a given phenotype (e.g., epidermal growth factor variant III, expressed in patients with glioblastoma multiforme and incapable of binding any known ligand [65]). However, they may vary between different types of tumor (e.g., heterogeneity of breast cancers, markers of which differ both among patients and within each individual tumor in one body [66]), but also changes related to the tumor progression. An exemplary case is metastatic tumors, as evolution of their genotypes occurs independently of the primary tumor and provides, inter alia, organ-specific adaptation to a new microenvironment. For instance, upregulation of L1 cell adhesion molecule (L1CAM) initiates outgrowths of colorectal cancer into perivascular sites [67]. All of the above might result in making some groups of cancer cells non-recognizable to a targeted carrier [68,69].

## 3. Composition of an Effective Lipopolyplex Based on Polyethyleneimine

The complexity of the structure of lipopolyplexes has some undeniable advantages. On the other hand, this fact is also responsible for complications during optimization of the process of its formation. First of all, a crucial factor to take into consideration is the N/P ratio, which corresponds to the molar ratio of, respectively, nitrogen atoms within positively charged imine groups of a polyethyleneimine to phosphorous atoms comprising anionic phosphate groups in a nucleic acid backbone. This impacts both the size of the resulting polyplexes and their net charge. Excess of PEI not only seems to prevent aggregation of such cationic polyplexes but might also improve transfection efficiency [70,71]. However, the optimal N/P ratio for efficient transfection is unique to each formulation, as it depends on various factors, such as size and topology of both of the polymers, ionic strength of the environment, and even the cell line to be transfected, some of which are discussed below [19,72].

Due to the electrostatic nature of the interactions between PEI and nucleic acids, their association, to a large extent, depends on the factors influencing the ionization of both molecules. For instance, it has been demonstrated that, in the case of 2.5 kDa linear polyethyleneimine (lPEI), both neutral pH and high ionic strength could lead to aggregation of its chains and precipitation, thus reducing the possibility of interaction with polyanionic molecules [73]. At the same time, it seems that the condensation efficiency might be improved by pre-heating the PEI solution to 60 °C before mixing it with DNA, consequently increasing the exposure of the imine groups of the polycation [74].

Another crucial interplay could be observed between polyplex formation and the molecular weight (MW) of the polyethyleneimine used. Hence, those with lower MW tend to exhibit poorer condensation of DNA and RNA, which can translate into diminished transfection using such complexes alone [70,75,76]. However, this tendency is reduced by enclosing polyplexes in liposomes, due to the lipid envelope properties [47,77]. Thus, the lipid composition seems to be, to a large extent, responsible for a carrier’s transfection efficiency [78]. It is even considered that, while, in the case of lipopolyplexes, PEI is mainly responsible for intracellular protection and distribution of nucleic acids, the lipid envelope plays an analogous role at the extracellular level. As a result, shorter chains of PEI are predominantly used for lipopolyplex formulations intended for in vivo administration, especially given their low cytotoxicity (the higher the MW, the more cytotoxic effect that polyplexes have) [75,79], whilst providing effective protection against nucleases [80,81].

It also seems that the topology of polyethyleneimine molecules is of considerable significance for the effective formation of polyplexes, despite the fact that currently no clear trend in this matter could be delineated. Namely, there are studies recognizing the superiority of linear PEI as a carrier of nucleic acids [70,82], as well as those favoring branched chains [83,84]. Most likely, it further depends on what size and kind of nucleic acid is condensed with a given PEI. For example, branched chains with high MW seem to work better with oligonucleotides, as opposed to 25 kDa lPEI, which is considered the gold standard when transfecting cells with plasmid DNA (pDNA). Interestingly, plasmid linearization hampers the complexation process [74,85,86]. Moreover, Kwok and Hart observed that pDNA complexes are more stable than those with siRNA, and the branched form of PEI is more effective in the case of RNA [87].

Next, the composition of the lipid envelope needs to be selected to a large extent with the aim of increasing, inter alia, the biocompatibility and stability of the carrier, hence the widespread use of glycerophospholipids with dominance of phosphatidylcholines and phosphatidylethanolamines with saturated acyl chains in their structure, which are able to spontaneously form a stable bilayer with low permeability at physiological temperature. In the case of lipopolyplexes, the most commonly used are: stability-enhancing cholesterol, phospholipids—anionic and zwitterionic ones and synthetic cationic lipids, examples and the structure of which are shown in Figure 1 [20,49,88,89,90,91,92,93]. Among these, particularly noteworthy is 1.2-dioleoyl-sn-glycero-3-phosphoethanolamine (DOPE), utilized both in cationic and neutral lipid shells. In the acidic environment of late endosomes, it is able to destabilize the membranes of these organelle and, thereby, partake in the release of the vector into the cytosol, helping to avoid its lysosomal degradation [94]. The displacement of cationic lipids, such as 1.2-dioleoyl-3-trimethylammonium-propane (DOTAP) or alkaline amino acid cholesterol derivatives (e.g., histidylated cholesterol), may be sometimes beneficial, because they exhibit more toxicity as they do not occur naturally in the body and could trigger an adverse interaction with serum and cell components due to the positive charge. However, various studies are still based on such positively charged carriers, due to their high transfection efficiency, as they are capable of electrostatic interaction with biological membranes, thus facilitating both the uptake of the vesicle and its subsequent release from the endosome [95,96,97,98]. It is further debated whether the addition of cationic lipids leads to their interaction with nucleic acids, resulting in further condensation [46,47,93,99].

**Figure 2:**
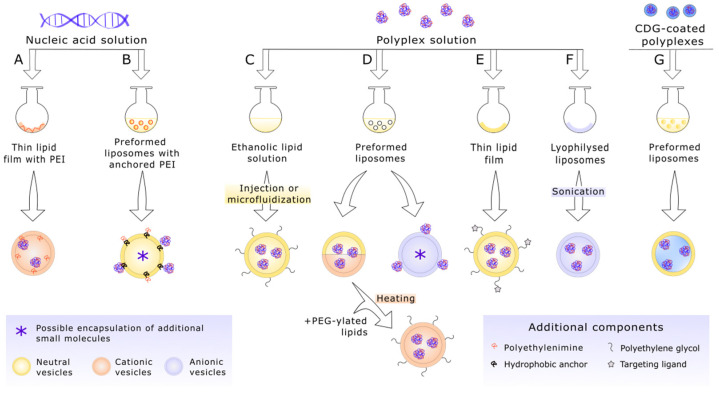
Possible procedures used in lipopolyplex preparation. A—Hydration of thin lipid film prepared from cationic lipids and polyethyleneimine (PEI) with nucleic acid solution [91]; B—reverse lipopolyplexes obtained through complexation of nucleic acid with PEI grafted to neutral lipid vesicles via hydrophobic anchor [110]; C—injection of polyplex solution into ethanolic solution of neutral lipids is a feasible way to obtain PEGylated lipopolyplexes [89,111]; D—incubation of polyplexes with preformed liposomes can result in either reverse lipopolyplexes (when anionic vesicles are used) [108,109] or “classic” lipopolyplexes (neutral or cationic vesicles) [47,49,54,58,88,91,93,96,98,102,103]; for preparation of cationic, stealth lipopolyplexes, PEGylated lipids could be incorporated in preformed carriers via mixture heating [96,98]; E—hydration of thin lipid film enables preparation of targeted lipopolyplexes [48,92]; F—hydration of lyophilized anionic liposomes might help polyplex internalization [88]; G—coating polyplexes with multicarboxyl dodecyl glucopyranoside anchors them to neutral lipid vesicles [40]. The presented approaches could be combined with each other and further modified, which enables a broad array of various lipopolyplexes to be generated.

Finally, the process of lipopolyplex formation itself seems to be equally important as the components; exemplary, commonly employed methods are presented in Figure 2. One of these is dry lipid film rehydration with a polyplex suspension, employed mainly when dealing with oligonucleotides. The utility of this technique, especially for more complex lipid formulations (e.g., immunolipopolyplexes), was demonstrated independently by Meissner et al. [48] and Ko et al. [92] and is still used in most recent studies concerning functionalized lipopolyplexes [64]. Meanwhile, Heyes et al. [89] proposed spontaneous vesicle formation, mixing the preformed polyplexes with an ethanolic lipid solution, which also turns out to be an efficient method of preparing PEGylated carriers for plasmid DNA. Penacho et al. [100] also stated that this method enables more robust transfection than with the hydration-extrusion method, when used to form lipopolyplexes with cationic lipids. Currently, some modification of this strategy comprising of the use of microfluidic technology or the dissolution of polyplexes in high-density polymers, such as poloxamer, have been demonstrated to increase the encapsulation efficiency [101].

Nevertheless, the technique that dominated the majority of published works relies on incubating liposome suspensions with already preformed polyplexes containing small RNA molecules, DNA oligonucleotides or plasmid DNA [47,49,54,58,88,91,93,96,98,102,103]. Presumably, the efficiency of such methods depends on exposure of vesicles to the fusogenic abilities of free polyethyleneimine chains and, in some cases, can be further enhanced by mechanical disruption of the lipid bilayer, e.g., via sonication. When using cationic liposomes, this strategy was demonstrated to be the most efficient one in comparison to mixing the cationic polymer with lipoplexes or with lipids and nucleic acid solutions simultaneously [104]. High transfection using lipopolyplexes obtained by this strategy was reported by Garcìa et al. [99], who carried out comparative tests on cationic liposomes encapsulating polyplexes. Interestingly, in this case, the order in which polymers were mixed together was also crucial; namely, addition of the branched PEI suspension to the DNA solution was found to be much more effective in terms of transfection than the other way around. This phenomenon was also reflected in the study of polyplexes alone conducted in vitro by Cho et al. [105]. The authors suggested that larger aggregates (about 200 nm), formed when PEI solution was instilled into DNA solution, are able to better overcome the barrier of lipid membranes. However, it is unknown whether, in the case of the experiments of Garcia et al. [99], this phenomenon might have translated into more effective internalization of polyplexes into liposomes. Even so, attention should be drawn to the possible dependence of the encapsulation efficiency using the presented method on the composition of the liposomes. Electron microscope observations confirmed that, in the case of an uncharged shell made of 1.2-dipalmitoyl-sn-glycero-3-phosphocholine (DPPC), this technique actually allows the encapsulation of polyplexes inside the vesicles [54]. It also seems that the efficiency of this process could be improved with the use of appropriate lipid anchors (e.g., by coating polyplexes with dodecyl-glucopyranoside derivative (CDG)) or by taking advantage of the electrostatic interactions between positively charged polyplexes and anionic lipids [40,88,93].

Even so, in most circumstances, there is still no clear evidence for complete internalization of complexes when incubating them with preformed liposomes. Thus, bearing in mind that Garcia et al. [99] only evaluated the effectiveness of the carrier based on the expression of the reporter gene, without explicitly estimating encapsulation efficiency of the polyplexes, it might be possible that the latter just associate with the carrier’s surface rather than migrate to the inner hydrophilic compartment of vesicles. Moreover, the presence of additional molecules on the surface, such as hydrophilic PEG, may constitute an additional barrier preventing the penetration of the complexes into the liposomes and/or their adsorption on the surface of such carriers. Hence, in some protocols, PEGylated lipids were added after the incubation of vesicles with polyplexes and subsequently heated to obtain surface-modified lipopolyplexes [96,98].

Despite the fact that most studies are aimed at polyplex encapsulation into liposomes, it does not seem to be necessary to obtain high transfection with simultaneous retained integrity of the therapeutic nucleic acids. Therefore, even just the conjugation of complexes of PEI and DNA or RNA to the surface of the vesicles seems to comply with these conditions [106,107]. Reverse lipopolyplexes, briefly mentioned earlier and illustrated in Figure 1, exactly fulfill the idea of transporting polyplexes as the external layer of the liposomal carrier. Exposure of polyethyleneimine helps to circumvent some limitations of lipid carriers, namely short circulation times (especially considering lipoplexes), simultaneously increasing endosomal release due to its increased efficiency as a proton sponge [86,95]. The standard procedure utilizes either non-covalent adsorption of polyethyleneimine to negatively charged lipids and surfactants constituting the preformed vesicles [108,109] or covalent modification of PEI with some hydrophobic anchor that enables close interaction with lipid bilayers. The reverse phase evaporation method is commonly used in such cases [110]. However, the protocol based on electrostatic interaction between PEI and the liposomal surface is heavily pH-dependent. As Sabín et al. [108] demonstrated, in the case of vesicles composed of zwitterionic lipids, association of PEI occurs only in a pH range providing opposite charges for both particles (e.g., for 1.2-dioleoyl-sn-glycero-3-phosphocholine (DOPC) liposomes, the optimal pH is in a range from 6 to 10). Thus, use of anchors, some of which additionally could facilitate nuclear transport (e.g., triamcinolone acetonide that acts as a nuclear localization signal) [110], might form more stable carriers.

## 4. Selected In Vivo Studies on Anticancer PEI-Based Lipopolyplexes

Recent preclinically in vivo tested therapies regarding lipopolyplexes focus mainly on silencing overexpressed and abnormal genes using miRNA, siRNA, and antisense oligonucleotides (ODN) [48,54,58,96,98,103] or the activation of suppressors using saRNA [88]. It is worth noting that these carriers, regardless of the type of nucleic acid, are in prevalence based on low-molecular, thus less toxic polyethyleneimines (2–10 kDa) and have a lipid shell composed of various phosphatidylethanolamines (PE) and phosphatidylcholines (PC). Another remarkable fact is feasibility of most of presented lipopolyplexes to intravenous administration (usually through tail vein in animal models), as it makes them adequate for fighting not only primary tumor but also metastasis. Exemplary formulations are summarized in Table 1.

Linder et al. [58] prepared vesicles with a diameter of around 300 nm and zeta potential close to zero, loaded with polyplexes based on Stat3-siRNA. In pathological conditions this protein is responsible for the activation of various genes that promote cancer, including those related to cell migration [113]. The group demonstrated extension of the life span of mice bearing glioblastoma, despite the fact that this locally administered formulation reached only a fraction of tumor cells. Ewe et al. [54], using the same phenomenon that is RNA interference but a different molecular target (survivin, an apoptosis inhibitor [114]), reduced PC-3 (prostate cancer) cell proliferation in a murine xenograft model, while observing a lack of immunostimulation upon systemic administration of DPPC-based lipopolyplexes. However, the excellent biodistribution of these non-PEGylated vesicles might result in part from intraperitoneal injection, rather than the more clinically relevant intravenous one.

In turn, our team [48] developed an antisense therapy based on vesicles decorated with the anti-CD20 antibody covalently bound to PEGylated lipids. Thanks to the active targeting strategy, this carrier could be utilized against both acute lymphoblastic leukemia and lymphomas, where CD20 is overexpressed on the surface of abnormal white blood cells. The approach chosen here was to decrease the expression of the anti-apoptotic BCL2 gene at the transcription level, which, in consequence, led to a reduction in the level of the cell survival-promoting protein, both in vitro and in NOD/SCID mice bearing xenograft tumors of Daudi human Burkitt’s lymphoma, after intravenous injection. Interestingly, the formulation stability was preserved for at least a year either in suspension or as freeze-dried powder.

An alternative approach was presented by Wang et al. [88], who utilized saRNA to stimulate the expression of the regulatory factor p21. This protein corresponds, inter alia, to cell cycle arrest in cells with damaged genetic material. Thanks to the additional coating with hyaluronic acid (HA), the anionic lipopolyplexes injected intratumorally were able to yield high transfection of colorectal cancer cells with abnormal amounts of CD44 receptor on their surface, since HA promotes adhesion and receptor-mediated endocytosis into such cells. This example represents another feasible strategy to limit cancerous cell division, leading, in consequence, to inhibition of tumor growth in an orthotropic xenograft model.

Xue et al. [40] designed a carrier that structurally mimics lentiviral particles, where a polyplex core is fused to the neutral vesicles using the aforementioned CDG derivative. Although, originally, the outer envelope consisted only of egg lecithin and cholesterol, it was demonstrated that it is possible to incorporate a variety of functional components in it, such as PEGylated lipids. Upon intravenous administration, the nanoparticles seemed to successfully deliver siRNA to the U87-MG glioblastoma cells implanted under the skin of nude mice, silencing the expression of vascular endothelial growth factor (VEGF), and thus reducing capillary density at the tumor site more efficiently than just polyplexes.

A slightly different approach was chosen by Jilek et al. [96] and Petrek et al. [98], who attempted enclosing small, non-coding RNA complexed with branched polyethyleneimine in a PEGylated shell containing cationic lipids. Both studies utilized recombinant miRNA molecules obtained via bacterial fermentation (BERA), that were based on a hybrid tRNA/pre-miRNA scaffold. The first of the mentioned approaches [96] introduced miRNA let-7c to mice bearing orthotopic hepatocellular carcinoma. This molecule, capable of inhibiting expression of the Bcl-xL protein, brings about apoptosis of the mentioned tumor cells in vivo, thus prolonging the life span of the tested animals. Meanwhile, the second team [98] applied an analogous strategy to the double hybrid let-7c/miR-124, controlling the expression of genes of the RAS, VAMP3, and CDK6 families, which prolonged the survival of non-small cell lung cancer xenograft in a murine model. Interestingly, in both studies, no significant changes of the markers (such as alanine aminotransferase, aspartate aminotransferase, albumin, creatinine, blood urea nitrogen, and total bilirubin) in the blood of the animals were detected, which was considered to be a hallmark of low toxicity of the preparations.

The complexity of pathological processes occurring in the cell during neoplasm also prompts the development of complex preparations with more than one molecular target. A remarkable example is the nanoparticles recently developed by Wang et al. [112] that carry simultaneously a plasmid containing the PTEN gene, an important suppressor of the cell cycle, and epigallocatechin gallate (EGCG), a flavonoid exhibiting strong antioxidant activity. The vesicles, containing the flavonoid in the hydrophilic core, additionally had 1.2-distearoyl-sn-glycero-3-phosphoethanolamine (DSPE) incorporated in the envelope conjugated with PEI chains projecting outwards, which enabled condensation of DNA on the carrier’s surface. This formulation takes advantage of EGCG’s ability to scavenge excess free radicals present in the tumor microenvironment, which may inactivate tyrosine phosphatases, such as PTEN. However, this flavonoid not only restores the functionality of the enzyme but also regulates other signaling proteins related to, for example, the apoptotic pathway [107]. Such multifunctional nanocarriers enabled the proliferation of PC-3 neoplastic cells to be limited in vitro, but also led to significant inhibition of tumor growth in vivo [112].

Another appealing example of bifunctional reverse lipopolyplexes was proposed by Mendes et al. [115]. This team developed bPEI-modified liposomes of egg PE and cholesterol that carry siRNA targeting multidrug resistance gene MDR1 and, subsequently, encapsulate chemotherapeutic drug—paclitaxel (PTX). The formulation takes advantage of synergy between PTX and siRNA-MDR1, as the latter downregulated proteins associated with drug resistance (such as P-glycoprotein), thus enhancing efficacy of PTX, a classic cancer drug that prevents microtubule disassembly. Moreover, such lipopolyplexes were able to promote in vivo tumor growth inhibition in an MDR xenograft ovarian tumor model (A2780-ADR cell line). This gives an exciting possibility for future combined therapies against drug-resistant cancers.

## 5. Possible Directions of Development of Lipopolyplexes

The necessity of constant improvement of a carrier’s pharmacokinetics and stability prompts scientists to test multi-component lipid envelopes [48,49]. Hence, increased attention is focused on the extracellular vesicles (ECVs) derived from various cell lines in vitro as an envelope for polyplexes [103]. Their similarity in terms of composition to natural cell membranes promises high immunotolerance and ease of crossing tissues and cell barriers, therefore making this carrier particularly suitable for intravenous administration [103,116]. Furthermore, it is suggested that uptake of such carriers bypasses the endosomal pathway, allowing their content to be delivered directly to the cytosol [103]. Zhupanyn et al. [103] presented ECV-modified PEI/survivin-targeting siRNA complexes that were able to inhibit prostate carcinoma xenografts in mice upon intravenous administration. The team harvested vesicles from various cell lines (SKOV, PC3) cultured in exosome-free medium and incubated them with polyplexes prior to ultrasound treatment. As observed, ECV derived from different cell lines were characterized by distinct surface markers, as well as exhibited a slight variation in transfection efficiency. However, despite the promising results obtained with this approach, there is a low probability of implementing it on an industrial scale in the near future, especially due to the complexity and high cost of production, as well as standardization problems.

A different formulation was proposed by Pinnapireddy et al. [117], who developed two novel envelopes for PEI-DNA lipopolyplexes, with composition mimicking HIV and HSV viruses. Lipids naturally occurring in viruses’ coating, such as DOPS, DOPE, and DPPC, conjugated with galactoside, were used with addition of cholesterol. The glycosylated lipid is especially interesting, as the sugar residues are known for prolonging the carrier’ stability in the bloodstream, i.e., being an alternative to polyethylene glycol coating. Such lipids might also have a positive effect on cellular uptake, as lipopolyplexes containing them would enter the cell via both clathrin- and caveolar-mediated endocytosis. Interestingly, HIV-mimicking carriers were less efficient than their HSV counterparts despite similar uptake mechanism. Definitely, further research is needed to establish behavior of such vesicles in vivo, as concerning hemolytic potential was observed by the authors.

Attempts to improve the biocompatibility and transfection efficiency of a polyethyleneimine-based carrier could also involve modification of the polymer’s chains. For polyplexes, various approaches have been studied to improve PEI behavior, including attachment of polyethylene glycol for prolonged retention in the blood, cyclodextrin for decreased cytotoxicity or fatty acids, such as oleic acid, for increased DNA binding capability of branched polyethyleneimine [118]. For lipopolyplexes, however, utilization of acrylate derivatives of linear PEI in combination with cationic lipid vesicles was proposed to achieve robust gene expression through enhancing the binding capacity of polyethyleneimine [95]. In another approach, cationic liposomes containing polyplexes based on pDNA and branched PEI conjugated with dexamethasone mesylate also exhibited elevated transfection levels in comparison to those with unmodified polyethyleneimine. This is most likely due to the ability of the synthetic glucocorticoid to dilate nuclear pores and ameliorate cytoplasmic trafficking [119].

An interesting strategy that is so far quite effective in regard to early generations of liposomal carriers is to employ stimuli that enable on-demand release of the cargo. This can be triggered by local differences characteristic for the tumor microenvironment [20,120]. An illustrative example is oxidation-sensitive lipopolyplexes based on branched polyethyleneimine modified with ferrocene-terminated alkyl chains and co-liposomes of lipopolymer and 1.2-dioleoyl-sn-glycero-3-phosphoethanolamine (DOPE) [106]. Lipopolyplexes with ferrocene, that carry DNA on their surface, in an oxidized state are internalized to a negligible degree, as opposed to when ferrocene is reduced. This makes it possible to regulate the expression of the introduced transgene by changing the reduction potential in the vicinity of the target cells. Moreover, such lipopolyplexes exhibit greater transfection efficiency in vitro than commercially available Lipofectamine 2000. However, further investigation on their in vivo activity and behavior is needed.

Another unusual approach focuses on improving the lipopolyplex cellular uptake via local use of low-frequency ultrasound (3 MHz) [121]. In in vitro studies such a procedure allows better penetration of the proliferating (outermost) layers of 3D spheroid culture of ovarian cancer SKOV-3 cells by vesicles carrying both an ultrasound contrasting agent (inside the carriers) and PEI-based polyplexes (present on the surface), even in the presence of endocytosis inhibitors. The approach also serves to increase the probability of the cargo’s release from the endosomes. The main limitation of this technique, however, is the dependence of its effectiveness on the application of topical ultrasound at an appropriate time point following cellular uptake, which would be extremely difficult to estimate if such a preparation was administered systemically in vivo.

In an alternative study, Chen et al. developed photoresponsive PEI-based lipopolyplexes to achieve light-triggered endosomal escape [122]. Verteporfin, a photosensitive molecule used commonly in photodynamic therapy, was incorporated into a bilayer formed of cholesterol, cationic lipids, and neutral PEGylated lipids. Then, lipopolyplexes were formed by rehydration of thin lipid film containing verteporfin with pDNA-bPEI polyplex solution. Upon light irradiation with a wavelength of 690 nm, the benzoporphyrin derivative generates reactive oxygen species that destabilize endosomes’ membranes which release their content to the cytoplasm. However, despite the harmlessness of the light stimulus alone, some concerns may be raised over application of such a strategy in anticancer therapies—first of all, the invasiveness associated with bringing an external light source to the tumor site, as well as the limited depth at which a given wavelength could penetrate tissues (in this case, up to 10 mm, which is not that deep considering larger tumor sizes).

Another remarkable attempt involved theranostic lipopolyplexes for noninvasive monitoring of the progress of implemented gene therapy by magnetic resonance imagining (MRI). Song et al. [123] developed MRI-recognizable liposome-polyethyleneimine complexes by first coating superparamagnetic iron oxide nanoparticles (SPION) with low molecular weight PEI, then injecting such suspension into ethanolic solution of DOTAP, DOPE, and cholesterol, and, finally, mixing slowly the obtained vesicles with plasmid DNA. However, in vitro studies on various cell lines (HepG2, SPC-A1, and A549) demonstrated low transfection efficiency of the proposed formulation, especially at high liposome/DNA ratio, indicating the need to further optimization of lipopolyplex composition and/or preparation process.

There are also some appealing, multifunctional carrier-based approaches, which combine variety of aforementioned strategies. For example, an active-targeting lipopolyplex developed by Samaddar et al. [111], that not only take advantage of PEI derivative, but could also serve as a platform for the subsequent transport of nucleic acids and small, hydrophobic drugs. In this approach, plasmid DNA (here, just carrying sequence encoding fluorescent protein EGFP as a marker) was condensed with a β-cyclodextrin-modified, low molecular weight (2.5 kDa) linear polyethyleneimine. Such alteration is supposed to reduce the cytotoxicity of PEI and facilitate the release of the cargo from endosomes. Additional incorporation of curcumin, a molecule known for inducing apoptosis and inhibiting proliferation of cancer cells, into the lipid envelope was possible by dissolving it together with lipids in ethanol, and then preparing lipopolyplexes by microfluidization. Furthermore, the authors used an unusual targeting strategy—the carrier was modified with a bacterial adhesin-mimicking RWFV peptide (amino acid sequence: GNRQRWFVVWLGSTNDPV) to its surface that could provide enhanced retention of the formulation in the bladder making it interesting for future in vivo studies.

One very compelling idea would be to test PEI-based lipopolyplexes as a delivery platform for components of the CRISPR/Cas system. Recently, there has been an abundance of research focused on novel delivery methods for this versatile and precise genome editing tool, as efficient delivery remains the main obstacle in utilizing it in gene therapies. For the time being, formation of stearyl octaarginine-based lipopolyplexes was proposed [124], although polyethyleneimine derivatives alone, as well as various liposomal or exosomal formulations, have been used for effective CRISPR/Cas system delivery, and they have been reviewed recently [125,126]. The next step would be now to combine both of those approaches together.

## 6. Summary

The preparation of polyethyleneimine-based lipopolyplexes is a multistage and fairly sensitive process, which additionally requires a specific balance to be maintained between its stability in the body, which would allow the appropriate dose of the preparation to reach the target site, and the ability to release nucleic acid at the right place and time. However, as the above examples of research show, such a carrier is a promising platform for future anti-cancer gene therapies, especially due to its versatility. The possibility to select from a broad array of components (DNA in the form of plasmid or oligonucleotides, small RNAs, polyethyleneimines of various length and topology, lipids, and/or additional targeting ligands) and exchange or modify them without affecting the overall carrier’s functionality makes it possible to tailor a lipopolyplex suitable for a given application. Moreover, lipopolyplexes’ performance in vivo is superior to bare polyplexes or nucleic acids, and, even though no formulation has yet reached the transfection effectiveness of viral carriers, they are able to safely deliver therapeutic nucleic acids, notwithstanding the manner in which these molecules are associated with the carrier (i.e., inside a lipid vesicle or on its surface). Finally, the repeatedly emphasized modularity and flexibility of lipopolyplexes allow considerable room for improvement of these carriers in the coming years.

## Figures and Tables

**Table 1:** Examples of PEI-based lipopolyplexes. BERA—bioengineered non-coding RNA agents, bPEI—branched polyethyleneimine, CDG-25 kDa PEI—25 kDa polyethyleneimine conjugated with multicarboxyl dodecyl glucopyranoside, Chol—cholesterol, DC-Chol—3β-[*N*-(*N*′,*N*′-dimethylaminoethane)-carbamoyl]cholesterol hydrochloride, DMG-PEG2000—1.2-dimyristoyl-rac-glycero-3-methoxypolyethylene glycol-2000 DOPE—1.2-dioleoyl-sn-glycero-3-phosphoethanolamine, DOTMA—1.2-di-O-octadecenyl-3-trimethylammonium propane, DPPC—1.2-dipalmitoyl-sn-glycero-3-phosphocholine, DSPE-PEG2000—1.2-distearoyl-sn-glycero-3-phosphorylethanolamine conjugated with 2000 kDa polyethylene glycol chains, DSPE-PEG-Mal—1.2-distearoyl-sn-glycero-3-phosphorylethanolamine conjugated with maleimide via polyethylene glycol chains, DSPE-PEI 25 kDa—1.2-distearoyl-sn-glycero-3-phosphorylethanolamine conjugated with 25 kDa polyethyleneimine, ECV—extracellular vesicles, HA—hyaluronic acid, HEPC—hydrogenated egg phosphatidylcholine, lPEI—linear polyethyleneimine, ODN—oligonucleotides, PC—phosphatidylcholine, pDNA—plasmid DNA, PE—phosphatidylethanolamine, PEI F25-LMW—low molecular weight, polyethyleneimine (4–10 kDa) derived through the fractionation of a commercially available 25 kDa branched PEI by size exclusion chromatography, saRNA—small activating RNA, siRNA—small inhibiting RNA.

Nucleic Acid	Polyethyleneimine	Lipids	Targeting Ligand	Administration Route	Targeted Cells	Mode of Action	Reference
saRNA	2 kDa bPEI	PE	HA	Local (injection to the rectum)	Human colorectal tumor xenografts	Stimulation of p21 expression	[88]
siRNA	PEI F25-LMW	DPPC	-	Intracranial	Tu2449 murine glioma cells	Knockdown of STAT3 gene expression	[58]
siRNA	PEI F25-LMW	DPPC	-	Intravenous	PC3 prostate carcinoma xenografts	Knockdown of survivin gene expression	[54]
siRNA	PEI F25-LMW	ECV	-	Intravenous	PC3 prostate carcinoma xenografts	Knockdown of survivin gene expression	[103]
ODN	2.5 kDa lPEI	HEPC, DOPE, DC-Chol, DSPE-PEG2000, DSPE-PEG-Mal	Anti-CD20 antibody conjugated via maleimide	Intravenous	Human Burkitt’s lymphoma Daudi cells	Reduction of the Bcl-2 protein level	[48]
pDNA	25 kDa bPEI	DPPC, Chol, DSPE-PEI25kDa	-	Intravenous	PC3 prostate carcinoma xenografts	Reacquisition of PTEN functionality	[112]
siRNA	CDG—25 kDa PEI	PC, Chol	-	Intravenous	U-87 MG glioblastoma xenografts	Silencing of VEGF expression	[40]
BERA	10 kDa bPEI	DOTMA, Chol, DMG-PEG2000	-	Intravenous	Hepatocellular carcinoma xenografts	Selective modulation of the expression of several genes (LIN28B, ARID3B, Bcl-xl, c-Myc)	[96]
				Intravenous	Non-small-cell lung carcinoma patient-derived xenografts	Selective modulation of the expression of several genes (RAS, VAMP3, CDK6)	[98]

## Data Availability

Not applicable.
